# Machine learning-enabled prediction of bone metastasis in esophageal cancer

**DOI:** 10.3389/fmed.2025.1620687

**Published:** 2025-06-30

**Authors:** Liqiang Liu, Wanshi Duan, Tao She, Shouzheng Ma, Haihui Wang, Jiakuan Chen

**Affiliations:** ^1^Tangdu Hospital, Fourth Military Medical University, Xi'an, China; ^2^Air Force Medical University, Xi'an, China

**Keywords:** esophageal cancer, bone metastasis, machine learning, XGBoost, prediction model

## Abstract

**Purpose:**

Bone metastasis (BM) is a common manifestation of distant spread in patients with esophageal cancer. This study aimed to develop a machine learning algorithm to predict the risk of bone metastasis in esophageal cancer patients, thereby supporting clinical decision-making support.

**Methods:**

Clinical and pathological data of esophageal cancer patients were obtained from the SEER database of the U.S. National Institutes of Health from 2010 to 2020. Six machine learning models were constructed: Support Vector Machine, Logistic Regression, Extreme Gradient Boosting, Neural Network, Random Forest, and k-Nearest Neighbors. Models performance was evaluated using accuracy, precision, recall, F1-score, and the area under the receiver operating characteristic curve. The optimal model was further used to interpret the associations between clinicopathological features and bone metastasis.

**Results:**

A total of 9,744 patients were included, with 532 (5.47%) had bone metastasis and 9,212 (94.53%) without. Multivariate logistic regression analysis identified age, T stage, N stage, and histological type as independent risk factors for bone metastasis. The XGBoost model demonstrated the best performance, achieving an accuracy of 0.80, a recall of 0.99, a precision of 0.72, an F1-score of 0.8300, and AUC of 0.92.

**Conclusion:**

The XGBoost model showed excellent predictive performance for bone metastasis in esophageal cancer patients, providing valuable insights for guiding clinical treatment decisions.

## 1 Introduction

Esophageal carcinoma (EC) is the seventh most commonly diagnosed cancer worldwide, with an estimated 604,000 new cases reported in 2020. It ranks sixth among all cancers in terms of mortality, accounting for approximately 544,000 deaths globally in the same year (1).

The incidence and histological subtypes of EC vary significantly across different geographic regions (2). EC primarily comprises two major histological subtypes: esophageal squamous cell carcinoma (ESCC) and esophageal adenocarcinoma (EAC). These subtypes differ markedly in terms of epidemiological patterns and biological behavior, making it essential to understand their distinctions for accurate diagnosis and effective treatment strategies (3). In the United States, individuals of White, Native American, and Black ethnicity are at a higher risk of developing EC compared to those of Hispanic or Asian ethnicity. Black patients are more likely to develop ESCC, whereas white patients are more commonly affected by EAC (4). Globally, EC imposes a significant disease burden, particularly in East Asia, Africa, and South America. ESCC originates from the stratified squamous epithelium of the esophagus and is ofen associated with chronic inflammation and mucosal injury, commonly occurring in the thoracic segment. Tobacco use and alcohol consumption are well-established risk factors for the development of invasive ESCC (5). In China, EC is the sixth most commonly diagnosed malignancy and the fourth leading cause of cancer-related mortality. Notably, approximately 90% of EC cases in China are ESCC, making it the predominant histological subtype both in China and globally (6). In contrast, esophageal adenocarcinoma is characterized by the malignant proliferation of glandular epithelial cells in the esophagus. The main risk factors include gastroesophageal reflux disease (GERD), Barrett’s esophagus (BE), tobacco abuse, obesity, and diets low in fruits and vegetables (7), In the United States, the incidence of EAC has been increasing and now accounts for over 60% of all esophageal cancer cases (8). Patients with EAC often present with more advanced T and N stage at diagnosis compared to those with ESCC. Despite the distinct etiological and pathological features of ESCC and EAC, their treatment approaches remained largely similar until recent advancements (9).

The 5-year survival rate for patients with metastatic esophageal cancer is extremely low, with only approximately 5% surviving beyond five years (10). Among distant metastatic sites, the liver is the most frequently involved, followed by the lymph nodes, lungs, bones, and brain. Interestingly, squamous cell carcinoma (SCC) tumors exhibit a higher rate for lung metastasis compared to adenocarcinoma (AC) subtype, whereas the AC subtype has a higher propensity for metastasis to the liver, bones, and brain (11). Developing predictive models to assess the risk and prognosis of bone metastasis in esophageal cancer is essential for guiding clinical management and improving patient outcomes.

Recent applications of machine learning in oncology have shown great promise in various domains, including medical image analysis, treatment planning, patient survival prognosis, and the synthesis of drugs at the point of care (12). However, limited research has focused on predictive models specifically targeting bone metastasis in esophageal cancer. This study aims to develop a machine learning algorithm for predicting the risk of bone metastasis in patients with esophageal cancer. We anticipate that such a predictive model will provide valuable insights to support clinical decision-making and ultimately improve patient outcomes.

## 2 Methods

### 2.1 Research design

**Figure 1:**
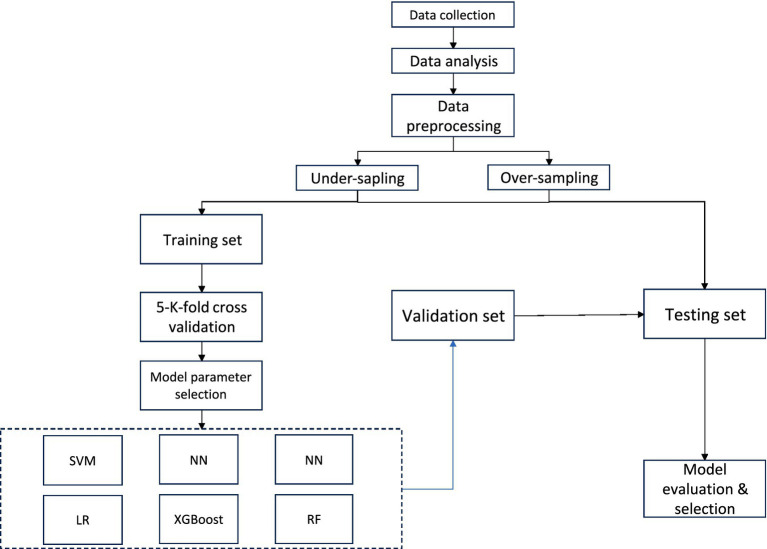
Research flowchart.

The software tools utilized in this study include Python version 3.8.0^1^ and SEER*Stat version.^2^ Patient data were extracted from the SEER database using SEER*Stat software. We included patients diagnosed with esophageal cancer (SCC and AC) between 2010 and 2020. The exclusion criteria were as follows: (1) patients with unknown brain, liver, or lung metastatic status; (2) patients with missing data on race race or histology grade; (3) patients with unknown primary tumor site; and (4) patients with incomplete T, N, or M stage information. A flowchart illustrating the case selection process is shown in Figure 1.

### 2.2 Data collection and clean

**Figure 2:**
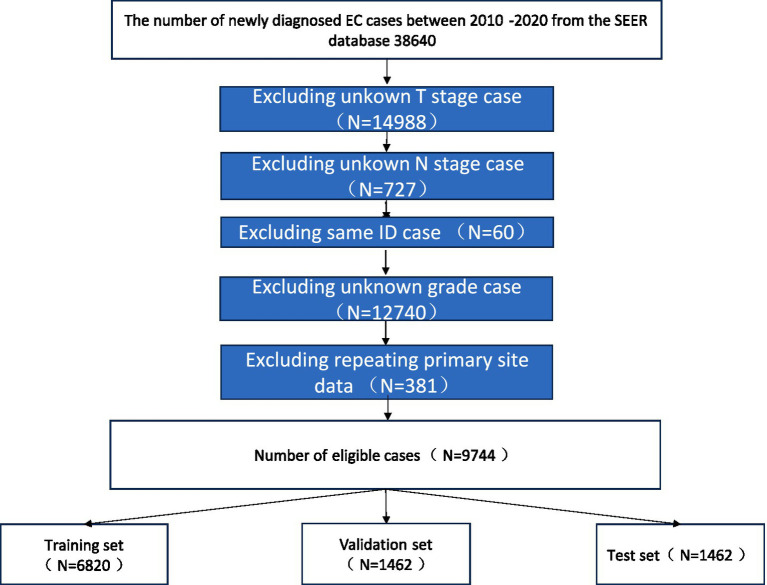
Data collection and clean.

In this study, 8 variables related to patients demographics and clinicopathological features were selected for analysis. The demographic variables included patient ID, age, sex, race, Clinicopathological variables included primary tumor site (site recode [ICD-O-3/WHO 2008], behavior code [ICD-O-3], tumor grade [grade thru 2017], grade pathological [2018+], tumor histology [ICD-O-3 Hist/behave], primary site-labeled, T stage, N stage, bone metastasis, all esophageal cancer patients were staged according the AJCC 7th and 8th edition guidelines and SEER staging information) (Figure 2).

### 2.3 Analysis of information

Significant variables among EC patients were initially identified through univariate logistic regression analysis (*p* < 0.05). Variables found to be statistically significant were subsequently included in a multivariate logistic regression analysis. Those that remained significant (*p* < 0.05) in the multivariate model were selected for further evaluation using machine learning models. Correlation analysis was performed to examine relationships among the selected features. Data preprocessing steps included label encoding to convert categorical text data into numerical format. Given the class imbalance due to the low incidence of bone metastasis (5.47%), the Synthetic Minority Over-sampling Technique (SMOTE) was applied to balance the dataset, resampling the minority class to achieve a 1:1 ratio (original distribution: 94.53% non-metastatic vs. 5.47% metastatic). The final balanced dataset was randomly partitioned into training (70%), validation (15%), and test (15%) subsets. The training set was used to fit the machine learning models, the validation set was employed for hyperparameter tuning and model selection, and the final test set was reserved for unbiased evaluation of predictive performance. To ensure the robustness of our results, model performance metrics such as AUC, precision, recall, and F1-score were calculated independently for each subset and are reported in the Results section.

### 2.4 The training set was used to develop six machine

Six machine learning models were employed in this study: Support Vector Machine (SVM), Logistic Regression (LR), Extreme Gradient Boosting (XGBoost), K-Nearest Neighbors (KNN), Random Forest (RF), and Neural Networks (NN). SVM is a binary classification algorithm that classifies data points by constructing an optimal hyperplane in a multidimensional space. LR evaluates the relationship between independent variables and the binary outcome variable, estimating the probability of an event based on a logistic function.

Following model implementation, performance was rigorously assessed using six metrics: accuracy, precision, recall, F1 score, AUC, and Brier score. The AUC, derived from the ROC curve, reflects the model’s diagnostic ability to distinguish between classes across various decision thresholds. The Brier score assesses the accuracy of probabilistic predictions and is particularly valuable in scenarios requiring probability-based classification. To further improve model performance, five-fold cross-validation and hyperparameter tuning were employed. GridSearchCV was utilized to systematically search for the optimal hyperparameter tunning, ensuring model optimization. Final model performance was determined by averaging the results across the cross-validation iterations.

### 2.5 Model interpretability

Interpreting machine learning models in an intuitive and clinical meaningful manner is essential for ensuring their practical applicability. To achieve this, a distribution plot of the target variable was generated to illustrate its original distribution concerning feature variables. Additionally, Partial Dependence Plots (PDPs) were constructed to visualize how individual feature variables influence the target variable and to analyze their impact on model predictions.

To further enhance model interpretability, SHapley Additive exPlanations (SHAP) values were calculated to quantify the contribution of each feature to the model’s predictions. A SHAP summary plot was generated to rank the feature importance and visualize their overall impact across all samples. SHAP dependence plots were used to examine the interaction between individual features and their corresponding SHAP values, providing deeper insights into how specific variables influence the model’s decision-making process.

The reliability of the model was evaluated by comparing the observed trends of the target variable with the predicted trends across different feature variables. This assessment integrated both PDPs and SHAP visualizations to ensure comprehensive and interpretable insights into model behavior.

## 3 Results

### 3.1 Analysis of information on EC patients

A total of 9,744 cases with EC were available, including 9,212 (94.53%) cases without bone metastasis and. 532 (5.47%) cases with bone metastasis, Age sex, histology, primary site, T stage, N stage, grade variables were significantly different between the two groups (all *p* < 0.05) Detailed information is summarized in Table 1.

**Table 1:** The detailed demographic information and pathological characteristics of the patients with EC.

Variable	Category	Total	No-bone metastasis	Bone metastasis	*p*-value
Age	<50	576	523 (90.97%)	53 (9.03%)	<**0.0001**
	50–60	1,870	1,752 (93.58%)	118 (6.42%)	
	60–70	3,376	3,188 (94.34%)	188 (5.66%)	
	70–80+	2,558	2,444 (95.39%)	114 (4.61%)	
	≥80	1,364	1,311 (96.26%)	53 (3.74%)	
Sex	Female	1,975	1,907 (96.56%)	68 (3.44%)	<**0.0001**
	Male	7,769	7,305 (94.03%)	464 (5.97%)	
Race	White	8,457	7,994 (94.53%)	463 (5.47%)	0.35
	Black	796	749 (94.10%)	47 (5.90%)	
	Asian or pacific islander	432	415 (96.06%)	17 (3.94%)	
	American Indian/Alaska native	59	54 (91.53%)	5 (8.47%)	
Histology	Adenocarcinoma	6,009	5,641 (93.88%)	368 (6.12%)	<**0.0001**
	Squamous cell carcinoma	2,881	2,776 (96.36%)	105 (3.64%)	
	Other carcinomas	854	795 (93.09%)	59 (6.91%)	
Primary Site-labeled	Cervical esophagus	227	218 (96.04%)	9 (3.96%)	0.04
	Upper third of esophagus	538	520 (96.65%)	18 (3.35%)	
	Middle third of esophagus	1,609	1,520 (94.47%)	89 (5.53%)	
	Lower third of esophagus	6,869	6,491 (94.50%)	378 (5.50%)	
	Abdominal/overlapping esophagus	501	463 (92.42%)	38 (7.58%)	
T stage	T1	2,972	2,798 (94.15%)	174 (5.85%)	<**0.0001**
	T2	1,274	1,242 (97.49%)	32 (2.51%)	
	T3	4,199	4,016 (95.64%)	183 (4.36%)	
	T4	1,299	1,156 (88.99%)	143 (11.01%)	
N stage	N0	4,120	4,002 (97.14%)	118 (2.86%)	<**0.0001**
	N1	4,112	3,810 (92.66%)	302 (7.34%)	
	N2	1,124	1,057 (94.04%)	67 (5.96%)	
	N3	388	343 (88.40%)	45 (11.60%)	
Grade	I	647	627 (96.91%)	20 (3.09%)	<**0.0001**
	II	4,143	3,983 (96.14%)	160 (3.86%)	

Bold *p*-values indicate statistical significance (*p* < 0.05).

Univariate analysis (Table 2) showed significant differences in the risk of bone metastasis across multiple variables, including age, sex, race, primary tumor site, T stage, N stages, tumor grade, and histology type (*p* < 0.05). Subsequently, multivariate logistic regression (Table 3) confirmed age, sex, T stage, N stage, and histology as independent prognostic factors for bone metastasis.

**Table 2:** Univariate analysis of variables related to bone metastasis.

Variable	Beta coefficient	95% CI	*p*-value
Age			
<50 years	2.31	(2.03 to 2.60)	<0.0001
50–60 years	0.37	(0.03 to 0.71)	0.03
60–70 years	0.50	(0.18 to 0.82)	0.0020
70–80 years	0.7188	(0.38 to 1.06)	<0.0001
≥80 years	0.94	(0.54 to 1.34)	<0.0001
Sex			
Female	3.33	(3.09 to 3.58)	<0.0001
Male	−0.58	(−0.84 to −0.32)	<0.0001
Race			
White	2.85	(2.76 to 2.94)	<0.0001
Black	−0.08	(−0.39 to 0.23)	0.61
Asian or Pacific Islander	0.35	(−0.15 to 0.84)	0.17
American Indian/Alaska native	−0.47	(−1.39 to 0.45)	0.32
Primary site			
Cervical esophagus	3.19	(2.52 to 3.85)	<0.0001
Upper third of esophagus	0.18	(−0.64 to 0.99)	0.67
Middle third of esophagus	−0.35	(−1.05 to 0.35)	0.33
Lower third of esophagus	−0.34	(−1.02 to 0.33)	0.32
Combined-abdominal	−0.69	(−1.43 to 0.06)	0.07
T stage			
T1	2.78	(2.62 to 2.93)	<0.0001
T2	0.88	(0.50 to 1.26)	<0.0001
T3	0.31	(0.10 to 0.52)	0.0042
T4	−0.69	(−0.92 to −0.45)	<0.0001
N stage			
N0	3.52	(3.34 to 3.7069)	<0.0001
N1	−0.99	(−1.2063 to −0.77)	<0.0001
N2	−0.77	(−1.07 to −0.46)	<0.0001
N3	−1.49	(−1.85 to −1.13)	<0.0001
Grade			
Well differentiated; grade I	0.23	(−0.24 to 0.70)	0.34
Moderately differentiated; grade II	3.21	(3.06 to 3.37)	<0.0001
Poorly differentiated; grade III	−0.64	(−0.83 to −0.45)	<0.0001
Undifferentiated; anaplastic; grade IV	−0.77	(−1.40 to −0.13)	0.02
Histology			
Adenocarcinoma	2.73	(2.62 to 2.84)	<0.0001
Other carcinomas	−0.13	(−0.41 to 0.16)	0.37
Squamous cell carcinoma	0.55	(0.32 to 0.77)	<0.0001

**Table 3:** Multivariate analysis of variables related to bone metastasis.

Variable	Coefficient	95% CI	*p*-value
Intercept	3.46	(2.52 to 4.40)	<0.001
Sex (female)	-	-	-
Male	−0.36	(−0.63 to −0.09)	0.01
Race (white)	-	-	-
Black	−0.32	(−0.67 to 0.02)	0.07
Asian or pacific islander	0.31	(−0.20 to 0.82)	0.24
American Indian/Alaska native	−0.36	(−1.31 to 0.59)	0.455
Primary site (cervical esophagus)	-	-	-
Upper third	0.04	(−0.79 to 0.87)	0.924
Middle third	−0.36	(−1.08 to 0.36)	0.332
Lower third	0.07	(−0.65 to 0.79)	0.853
Combined abdominal	−0.19	(−0.97 to 0.59)	0.637
T stage (T1)	-	-	-
T2	1.07	(0.68 to 1.46)	<0.001
T3	0.76	(0.52 to 0.99)	<0.001
T4	−0.28	(−0.52 to −0.03)	0.03
N stage (N0)	-	-	-
N1	−1.00	(−1.23 to −0.77)	<0.001
N2	−0.92	(−1.24 to −0.59)	<0.001
N3	−1.36	(−1.75 to −0.97)	<0.001
Grade (well differentiated, grade i)	-	-	-
Moderately differentiated, grade ii	−0.18	(−0.66 to 0.30)	0.47
Poorly differentiated, grade iii	−0.68	(−1.15 to −0.21)	0.004
Undifferentiated, grade IV	−0.78	(−1.56 to 0.01)	0.05
Age (<50 years)	-	-	-
50–60 years	0.26	(−0.09 to 0.61)	0.14
60–70 years	0.34	(0.01 to 0.67)	0.04
70–80 years	0.45	(0.10 to 0.80)	0.01
≥80 years	0.62	(0.21 to 1.04)	0.003
Histology (adenocarcinoma)	–	–	–
Squamous cell carcinoma	0.67	(0.38 to 0.97)	<0.001
Other carcinomas	0.15	(−0.15 to 0.45)	0.32

### 3.2 Spearman’s correlation and feature importance

**Figure 3:**
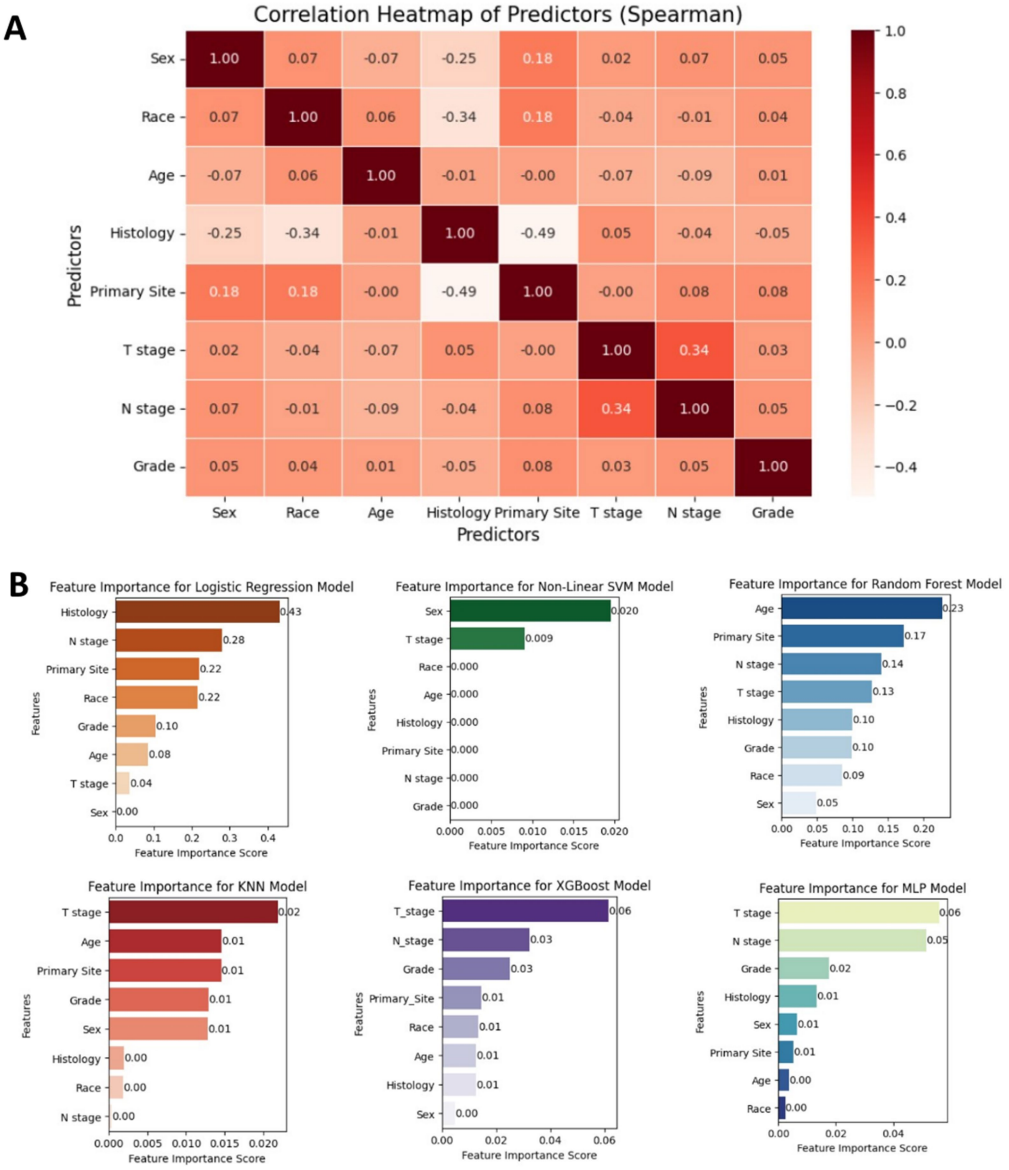
**(A)** Spearman correlation heatmap displaying the relationships among key clinicopathological variables used in the model, indicating minimal multicollinearity. **(B)** Bar plot showing the relative importance of each feature across the six machine learning models. T stage and N stage were consistently among the top contributors to model performance.

To evaluate the strength of relationships among variables, correlation analysis was conducted. Specifically, Spearman’s rank correlation analysis was employed to evaluate the correlations among the selected features. As shown in Figure 3A, the resulting heatmap demonstrated a lack of strong correlations among the eight analyzed variables, indicating low multicollinearity. Figure 3B presents the feature importance extracted from each machine learning algorithm. Variables identified via univariate and multivariate logistic analyses all played significant roles in predicting the outcomes of the six models. Notably, T stage has consistently been the most influential feature in most prediction models, emphasizing its critical impact on bone metastasis in esophageal cancer. The eight features of the XGBoost model are ranked from high to low importance.

### 3.3 Interpretability of the model

Among all models, XGBoost demonstrates the best performance, achieving the highest AUC and sensitivity, along with relatively superior values in other evaluation matrics. Therefore, XGBoost was identified as the optimal predictive model for the current dataset (see Table 4).

**Table 4:** Predictive performance of different models.

Model	Accuracy	Precision	Sensitivity	F Score	AUC
LR	0.59	0.59	0.61	0.60	0.62
SVM	0.79	0.77	0.84	0.80	0.85
RF	0.81	0.78	0.87	0.82	0.90
KNN	0.69	0.85	0.47	0.61	0.80
XGBoost	0.80	0.72	0.99	0.83	0.92
NN	0.81	0.78	0.88	0.82	0.89

**Figure 4:**
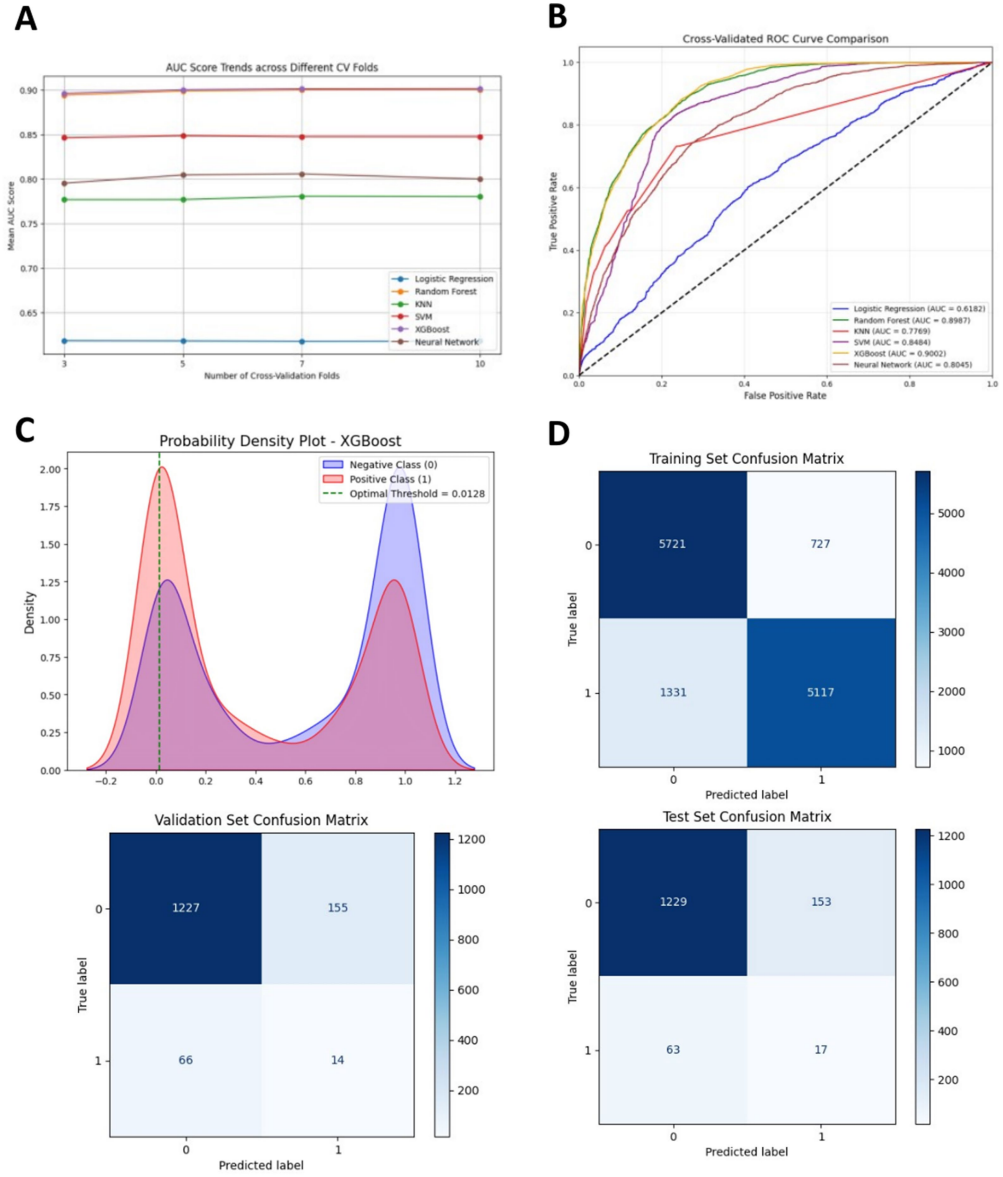
**(A)** Receiver Operating Characteristic (ROC) curves of different machine learning models in the internal test set. **(B)** Five-fold cross-validation results of different machine learning models. **(C)** Probability density plot of the XGBoost model. **(D)** Confusion matrices of the XGBoost model in the training set and the internal test set. TP represents true positive, TN represents true negative, FP represents false positive, and FN represents false negative.

The performance of the six prediction models is shown in Figures 4A,B, and Table 3. The internal 5-fold cross-validation (Figure 4A) reveals that among models, XGBoost model demonstrates the best performance, with an average AUC of 0.90. The RF model ranks second (AUC = 0.90). The internal test validation results are presented in Table 3 and Figure 4B. Notably, the XGBoost model also achieves the highest AUC score in the internal test validation (AUC = 0.92), with recall and F1 scores of 0.99 and 0.83, respectively. The confusion matrices of the XGBoost model on the training and test sets (Figure 4C) further highlight its high recall. The probability density plot of predictions (Figure 4D) indicates that the AUC reaches its maximum value when the prediction score is set at 0.01.

**Figure 5:**
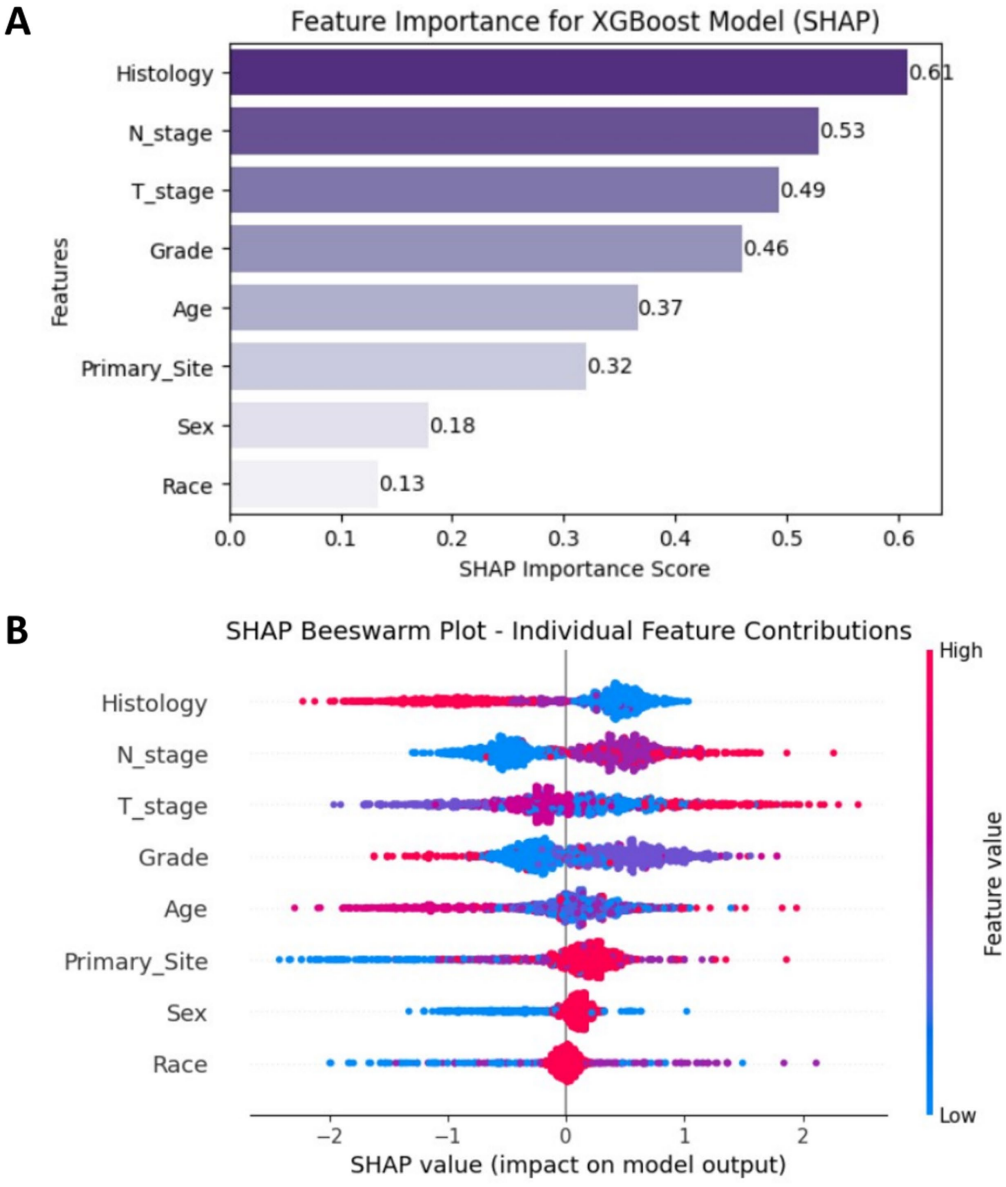
**(A)** SHAP feature importance plot ranked by mean absolute values. **(B)** SHAP beeswarm plot showing individual sample contributions.

The SHAP analysis results of the XGBoost model are presented as follows, Feature importance analysis (Figure 5A) showed that among the eight features, Histology (mean SHAP value = 0.61), N stage (0.53), and T stage (0.49) were the top three contributors in terms of mean absolute SHAP values. These findings indicated that Histology type, N stage, and T stage had the most substantial influence on model predictions, underscoring their critical roles in predicting bone metastasis in EC. Individual feature contribution analysis (Figure 5B) revealed distinct patterns: features like N stage and T stage demonstrated clear trends where higher feature values (represented by red dots) positively contributed to the prediction of bone metastasis. In contrast, Sex and Race had minimal impacts, as their SHAP values clustered near zero, indicating negligible contributions to the model’s output. Overall, the SHAP analysis not only quantified the relative importance of clinical features but also provided intuitive visualizations of how each feature affected model predictions, offering a theoretical foundation to support clinical decision-making in the context of bone metastasis in esophageal cancer.

## 4 Discussion

Distant metastasis remains the primary cause of treatment failure and mortality in EC (13). Bone metastasis (BM) is the third most common site of distant spread in EC and is associated with significantly worse survival outcomes. Patients with BM have been reported to experience the poorest prognosis among those with metastatic (11). In our study, the incidence of BM among EC patients was 5.4%, which is consistent with previous reports ranging from 5.2 and 7.7%. Given the overall poor prognosis of EC, early identification of high-risk factors and the development of reliable predictive models for BM based on clinical and pathological characteristics are crucial for guiding individualized treatment strategies and improving clinical decision-making.

In our study, 75% of EC patients were over the age of 60. Interestingly, older patients exhibited a lower probability of BM, a finding consistent with the study by Yuan et al. and Qin et al., which identified a higher BM risk in patients aged 51–60 compared to those aged 71–80 (14). This phenomenon may be attributed to capillary sclerosis, which could reduce the likelihood of distant metastasis in older adults (15).

Our study aligns with the findings of Hayam et al. (16), showing that approximately 80% of EC patients are male. In our study, male patients demonstrated a significantly higher incidence of BM than females. This discrepancy may be attributed to behavioral and hormonal differences; men are more likely to smoke and consume alcohol, both of which are established EC risk factors (17). Additionally, male sex hormones have been implicated in promoting EC cell proliferation and metastasis (18), Differences in musculoskeletal health and sex hormone levels may also contribute to these variations (19). Our findings also suggest that metastatic male EC (MEC) patients have a higher incidence of bone-only metastasis compared to metastatic female EC (FEC) patients (17).

Consistent with previous studies, adenocarcinoma (AC) was the predominant histological subtype in our cohort. AC demonstrated a higher tendency for metastasis to the liver, bones, and brain compared to squamous cell carcinoma (SCC) (20). These findings reinforce the notion that EC subtypes exhibit distinct metastatic patterns due to differences in tumor origin, pathogenesis, and anatomical distribution (21). However, in contrast to earlier studies, we did not observe significant differences in lymph node and BM rates between SCC and AC. This discrepancy may be explained by the fact that previous studies primarily included stage IV patients, which could limit the observation of bone metastases (11). While it is widely recognized that advanced T and N stages are associated with an increased risk of bone metastasis, our multivariate analysis unexpectedly revealed that patients with N3-stage disease had a lower risk of developing BM. This paradoxical finding may be explained by the shorter survival time of N3 patients, which may prevent the progression to bone metastasis before death.

With the rapid advancements of artificial intelligence, machine learning (ML) has shown great promise in biomedical applications, including EC diagnosis and prognosis prediction (22–24). Previous studies have primarily focused on predicting liver and lung metastases in EC. To our knowledge, this study is the first to construct an ML-based predictive model for BM in EC using the SEER database, Yuan et al. (14) developed a predictive nomogram for BM in EC patients, reporting AUC values of 0.77 and 0.75 in the training and validation cohorts, respectively. In contrast, our ML model specifically designed for BM prediction and utilized a larger sample size (*n* = 9,744), thereby improving statistical power and model robustness. The XGBoost algorithm, which has demonstrated high accuracy and ease of use in various studies (25, 26) exhibited superior performance in our study. Our XGBoost model achieved outstanding predictive accuracy (AUC = 0.92, recall = 0.98), surpassing traditional models, same as recent ML-based liver metastasis prediction models (AUC = 0.92) (27). The high recall rate (98%) suggests that our model effectively identifies high-risk BM patients, minimizing the likelihood of missed diagnoses.

Additionally, ML models provide valuable insights into the complex relationships among independent prognostic factors-an aspect often overlooked in conventional statistical analyses. While multivariate logistic regression and Cox regression identified certain risk factors, but some of these variables had negligible SHAP values in feature importance rankings. This discrepancy highlights the advantage of ML, as it eliminates irrelevant features and reduces the risk of overfitting, unlike traditional regression models. Furthermore, ML continuously improves operational efficiency and predictive accuracy through self-learning mechanisms.

Despite the robustness of our findings, several limitations should be acknowledged. Given the ethnic and regional differences in EC incidence-particularly the high prevalence in East Asia-future studies should include large-scale external validation using datasets from Chinese or other East Asian patient populations to enhance the generalizability and applicability of the predictive model.

Despite the relatively low incidence of bone metastasis in esophageal cancer, its profound prognostic implications and association with significant morbidity justify the need for risk stratification tools. We emphasize that the primary goal of our model is not to replace clinical judgment but to provide an adjunctive decision-support mechanism. By identifying high-risk individuals early-especially in cases with atypical or silent presentations-the model has the potential to inform more personalized surveillance strategies, improve resource allocation, and ultimately contribute to better clinical outcomes.

## 5 Conclusion

In summary, this study presents the first ML-based predictive model for BM in EC using the SEER database, providing a valuable tool for precision oncology. Future research should focus on cross-ethnic validation, multi-modal data integration, and explore translational applications to establish a clinically actionable predictive-to-preventive continuum.

## Data Availability

The original contributions presented in the study are included in the article/supplementary material, further inquiries can be directed to the corresponding author.

## Footnotes

^1^ https://www.python.org/

^2^ https://seer.cancer.gov/seerstat/
